# Inflammatory cytokines may mediate the causal relationship between gut microbiota and male infertility: a bidirectional, mediating, multivariate Mendelian randomization study

**DOI:** 10.3389/fendo.2024.1368334

**Published:** 2024-04-22

**Authors:** Haoxi Zou, Ningning Xu, Huanying Xu, Xiaoyan Xing, Yanfen Chen, Suzhen Wu

**Affiliations:** ^1^ Foshan Clinical Medical School of Guangzhou University of Chinese Medicine, Foshan, China; ^2^ TCM Gynecology Department, Foshan Fosun Chancheng Hospital, Chancheng District, Foshan, China

**Keywords:** gut microbes, inflammatory cytokine, male infertility, Mendelian randomization, causal inference, genetics

## Abstract

**Introduction:**

Studies have shown that the gut microbiota is associated with male infertility (MI). However, their causal relationship and potential mediators need more evidence to prove. We aimed to investigate the causal relationship between the gut microbiome and MI and the potential mediating role of inflammatory cytokines from a genetic perspective through a Mendelian randomization approach.

**Methods:**

This study used data from genome-wide association studies of gut microbes (Mibiogen, n = 18, 340), inflammatory cytokines (NFBC1966, FYPCRS, FINRISK 1997 and 2002, n=13, 365), and male infertility (Finngen, n=120, 706) to perform two-way Mendelian randomization (MR), mediated MR, and multivariate MR(MVMR) analyses. In this study, the inverse variance weighting method was used as the primary analysis method, and other methods were used as supplementary analysis methods.

**Results:**

In the present study, two gut microbes and two inflammatory cytokines were found to have a potential causal relationship with MI. Of the two gut microorganisms causally associated with male infertility, Anaerotruncus increased the risk of male infertility (odds ratio = 1.81, 95% confidence interval = 1.18-2.77, P = 0.0062), and Bacteroides decreased the risk of male infertility (odds ratio = 0.57, 95% confidence interval = 0.33-0.96, P = 0.0363). In addition, of the two inflammatory cytokines identified, hepatocyte growth factor(HGF) reduced the risk of male infertility (odds ratio = 0.50, 95% confidence interval = 0.35-0.71, P = 0.0001), Monocyte chemotactic protein 3 (MCP-3) increased the risk of male infertility (odds ratio = 1.28, 95% confidence interval = 1.03-1.61, P = 0.0039). Mediated MR analysis showed that HGF mediated the causal effect of Bacteroides on MI (mediated percentage 38.9%). Multivariate MR analyses suggest that HGF may be one of the pathways through which Bacteroides affects MI, with other unexplored pathways.

**Conclusion:**

The present study suggests a causal relationship between specific gut microbiota, inflammatory cytokines, and MI. In addition, HGF may mediate the relationship between Bacteroides and MI.

## Graphical Abstract

Bidirectional causality between gut flora, inflammatory cytokines and male infertility. 119 bacterial taxa contain 119 species of gut microorganisms at the genus level. The 41 inflammatory cytokines contain pro-inflammatory and anti-inflammatory cytokines.

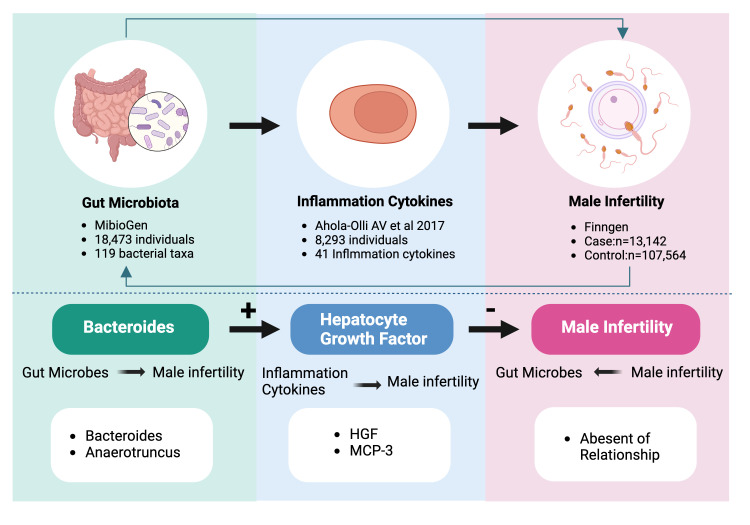

## 1 Introduction

Infertility is afflicting 9% of couples worldwide. According to World Health Organization estimates, male infertility accounts for about 30-50% of infertility cases (1). There are various causes of male factor infertility, including congenital, acquired, idiopathic, or environmental factors (2), such as genetic alterations, microbiome changes, and the impact of environmental pollutants (3, 4).

Gut microbiota refers to the community of microorganisms that reside within the human digestive system, including various microorganisms, including bacteria, fungi, and viruses. These microorganisms are important role in human health and immune regulation (5). Recent studies have found a causal link between gut microbiota and male infertility. Evidence suggests that immune system activation due to gut microbiota translocation leads to testicular and epididymal inflammation and induces insulin resistance, which in turn affects sex hormone production and regulates spermatogenesis (6, 7). It has been established that the gut microbiota plays crucial roles in spermatogenesis and male fertility, as demonstrated by various animal modeling experiments (7–9). In addition, a prospective clinical trial to compare the taxonomic and functional profiles of the gut, semen, and urine microbiomes of infertile and fertile men (10). However, The relevance of animal models can be limited in mimicking human pathophysiology (11). Moreover, implementing the prospective clinical trial was challenging due to multiple constraints in the clinical setting, and the sample size was small. The Mendelian Randomization study method is a powerful tool in epidemiological research, utilizing genetic variation as to assess the causal association between risk factors and specific diseases (12). Previous Mendelian Randomization studies (13–15) found a causal correlation between gut microbiota and male infertility. However, these studies only focused on exploring whether there is a causal relationship between specific gut microbiota and the risk of MI, without investigating the potential mediating factors and mechanisms of disease development between them.

Inflammatory cytokines are important mediators between activated immune and non-immune cells, including epithelial and mesenchymal cells (16). They are immunomodulatory peptides released by many cells and have a significant impact on immune function. They can be biomarkers to indicate or monitor disease or its progression and may serve as clinically applicable therapeutic parameters (17–19). Interestingly, testicular macrophages have been implicated in the pathogenesis of various inflammatory diseases (20), underscoring inflammation as a fundamental causative factor in male infertility (21). Epidemiological studies consistently demonstrate that men who have infertility often exhibit chronic inflammation of the male reproductive tract, which in turn, exacerbates fertility issues (22). Experimental models of autoimmune orchitis have further elucidated this relationship, revealing substantial macrophage infiltration and the secretion of numerous inflammatory factors, thereby highlighting the intricate interplay between inflammation and male reproductive health (23, 24).

When gut microbiota is dysregulated, it increased secretion of pro-inflammatory factors and activates macrophages and dendritic cells in the testis (25). Upon entering the epididymis, these innate immune cells may recognize sperm as foreign substances and attack them, affecting their survival and function. Additionally, the gut microbiota synthesizes short-chain fatty acids (SCFAs), notably butyrate, which are crucial in guiding the differentiation of peripheral naive CD4+ T cells outside the thymus into Foxp3+ Treg cells (26). This process fosters the development of M2-type macrophages and plays a significant role in dampening inflammation (27). SCFAs achieve this by inhibiting the generation of nitric oxide and releasing of pro-inflammatory cytokines, such as TNF-α, IL-1β, and IL-6, in macrophages activated by lipopolysaccharides. Simultaneously, SCFAs enhance the secretion of the anti-inflammatory cytokine IL-10, thereby obstructing the NF-κB signaling pathway (28). Moreover, the upregulation of inflammatory cytokines is linked with the onset of insulin resistance (29), adding another layer to the complex influence of gut microbiota on male reproductive health.

Based on these, we hypothesized that inflammatory factors mediate the effects of gut microbiota on male infertility. Therefore, we conducted a Mendelian randomization study to explore whether inflammatory factors mediate effect in this potential causal relationship. With this study design, we expect to reveal the specific roles of inflammatory factors in the gut flora influencing male infertility, thus providing a scientific basis for the developing targeted therapeutic strategies. This will not only enhance our understanding of the relationship between gut microbes and male reproductive health but may also provide new ideas for the treatment of male infertility, especially for those patients who have had limited success with conventional treatments. In addition, this line of research also emphasizes the importance of inflammatory factors as potential therapeutic targets, prompting us to explore in depth the role of inflammation in the pathogenesis of male infertility and providing new strategies and directions for clinical treatment.

## 2 Methods

### 2.1 Study design

**Figure 1:**
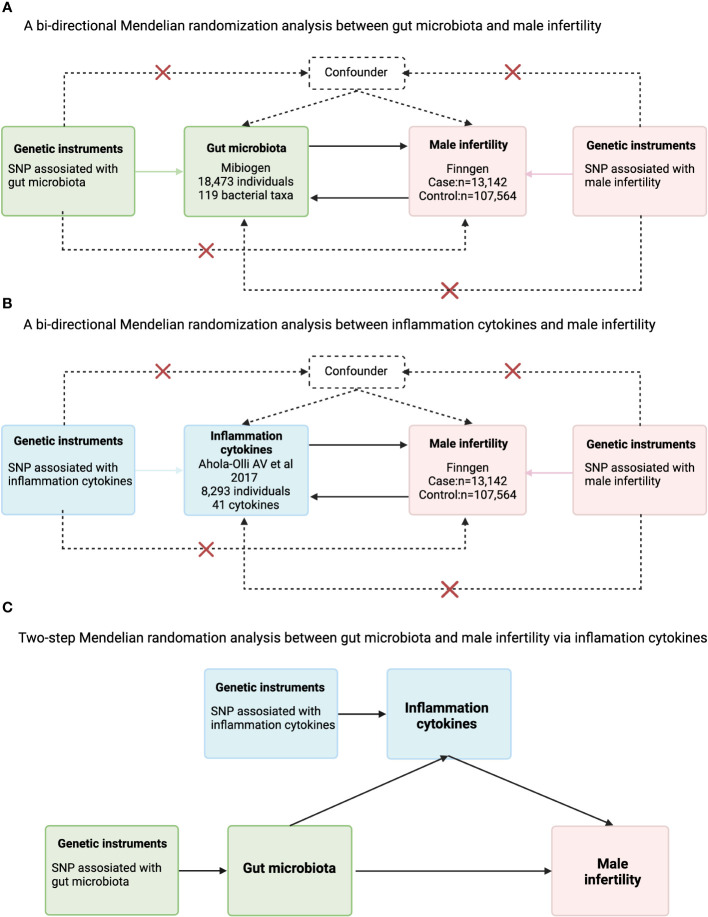
Flowchart for the analysis of Mendelian randomization of inflammatory cytokines that may act as mediators between gut microbes and male infertility. **(A)** A bi-directional Mendelian randomization analysis between gut microbiota and male infertility; **(B)** bi-directional Mendelian randomization analysis between inflammation cytokines and male infertility; **(C)** Two-step Mendelian randomation analysis between gut microbiota and male infertility via inflammation cytokines.

This MR study was divided into two phases(The flowchart is depicted in **Figure 1**). In the first phase, we first investigated the causal effect of 119 species of gut flora at the Genus level and male infertility. In the second phase we assessed the mediating role of inflammation cytokines in the causal relationship between gut flora and male infertility. Our MR analyses needed to satisfy the following three major assumptions: (1) instrumental variables (IVs) are associated with exposure (correlation assumption); (2) IVs are not associated with confounders (independence assumption); (3) IVs affect outcomes only through exposure (exclusivity assumption). Because genetic variants form randomly at conception according to Mendelian laws of inheritance, the results of MR analysis are unlikely to be affected by confounding factors and reverse causality. We wrote this study based on the Report on Strengthening Observational Studies in Epidemiology Using Mendelian Randomization Methods (STROBE-MR). Summary data from genome-wide association studies (GWAS) were used in this study, targeting mainly Europeans from professional organizations or research. **Supplementary Table 1** lists information about the GWAS datasets used in this study.

### 2.2 Data sources

IVs for gut microbiota (n=18340) were derived from the largest GWAS abstract data to date from the MiBioGen consortium. The study population was 18340 individuals from 24 cohorts of European, Spanish, Middle Eastern, Asian, and African ancestry (30). GWAS summary data on circulating levels of inflammatory cytokines utilized samples from 13365 Finns from the Northern Finland Birth Cohort 1966 (NFBC1966), the Finnish Young People’s Cardiovascular Risk Study (FYPCRS), and FINRISK 1997 and 2002. As a genetic tool for male infertility, we used GWAS summary data from the Finngen Consortium from 120706 European individuals (case = 13142, control = 107564) (31).

### 2.3 IVs selection

We used the genetic variation of microbiota taxa by GWAS test P-value threshold (<5 × 10^-6^). We used the same threshold (<5 × 10^-6^) during the selection of genetic instrumental variables for inflammatory cytokines. We then clustered all these genetic variants to a chain disequilibrium threshold of R_2_ < 0.001 within a distance of ± 5000 kb using the 1000 Genomes European reference panel, respectively. A crucial phase in Mendelian Randomization (MR) analysis involves verifying that the impact of Single Nucleotide Polymorphisms (SNPs) on the exposure is aligned with the same allele as their effects on the outcome. Upon aligning the outcome data, we excluded palindromic SNPs to maintain accuracy. (Palindromic SNPs are those with alleles of either A/T or G/C, where the forward and reverse sequences are identical.) We extracted pertinent data including chromosome location, effect allele (EA), other allele (OA), effect allele frequency (EAF), effect sizes (β), standard error (SE), and P-value. Subsequently, we calculated the explained variance (R^2^) and F-statistic to evaluate the strength of association between the identified instrumental variables (IVs) and the exposure. Typically, SNPs with an F-statistic below 10 are considered weak instruments (32). In this study, R^2^ = 2 × EAF × (1-EAF) × β^2^/(2 × EAF × (1-EAF) × β^2^ + 2 × EAF × (1-EAF) × N × SE^2^), where N is the sample size of the GWAS, and F = R^2^ × (N-2)/(1-R^2^) (33).

### 2.4 Statistic analysis

The inverse variance weighting (IVW) method was used as the main method for causality estimation, which combines the Wald ratios of individual SNPs to assess outcomes. We calculated Cochrane’s Q-derived P-values to assess the degree of heterogeneity. In addition, we used MR-Egger and weighted median for sensitivity analysis. We also used the MR multivariate residuals and outliers (MR-Presso) method to detect possible outliers and to calculate causal estimates after removing identified outliers (34).

We performed a two-step MR analysis to determine the mediating effect of inflammatory cytokines on the relationship between gut microbiota and male infertility. The proportion of inflammation cytokines in the total effect was estimated by dividing the indirect effect by the total effect (β1 × β2/β3), with β1 representing the effect of gut microbiota on inflammatory cytokines, β2 representing the effect of inflammatory cytokines on MI, and β3 representing the effect of gut microbiota on MI. Standard errors were derived by bootstrap and effect estimates by two-sample MR analysis.

In addition, we performed an MVMR to analyze whether a causal relationship between the gut microbiota and male infertility still exists after modulating the inflammatory cytokines, and to calculate the direct effect of the gut microbiota on male infertility.

In this study, the results of MR analysis for binary outcomes are presented using odds ratio(OR), where an OR greater than 1 indicates a higher risk of disease, and an OR less than 1 indicates a lower risk of disease. For continuous outcomes, the results are presented using Beta, where the sign of the Beta value indicates the direction of association. A positive Beta value means that as the level of exposure increases, the outcome variable (either levels of inflammatory markers or disease risk) also increases; a negative value suggests that an increase in exposure level leads to a decrease in the outcome variable. We did not correct for multiple testing in this exploratory study.

All analyses were performed on the R platform (version 4.2.2). Statistical analyses and data visualization were performed using the “TwoSampleMR”, “Mendelian Randomization”, “ggplot2”, “RMediation” and “MVMR” software packages.

## 3 Results

The SNPs used for MR analysis in the three GWAS data for gut microbiota, inflammatory cytokines, and male infertility are shown in **Supplementary Tables 2** and **3**, respectively. The F-statistics of all SNPs used for analysis were greater than 10. Sample overlap was <1% for both GWAS data on gut microbiota/inflammatory cytokines and MI (**Supplementary Table 1**).

### 3.1 Casual relationship between microbiota and male infertility

**Figure 2:**
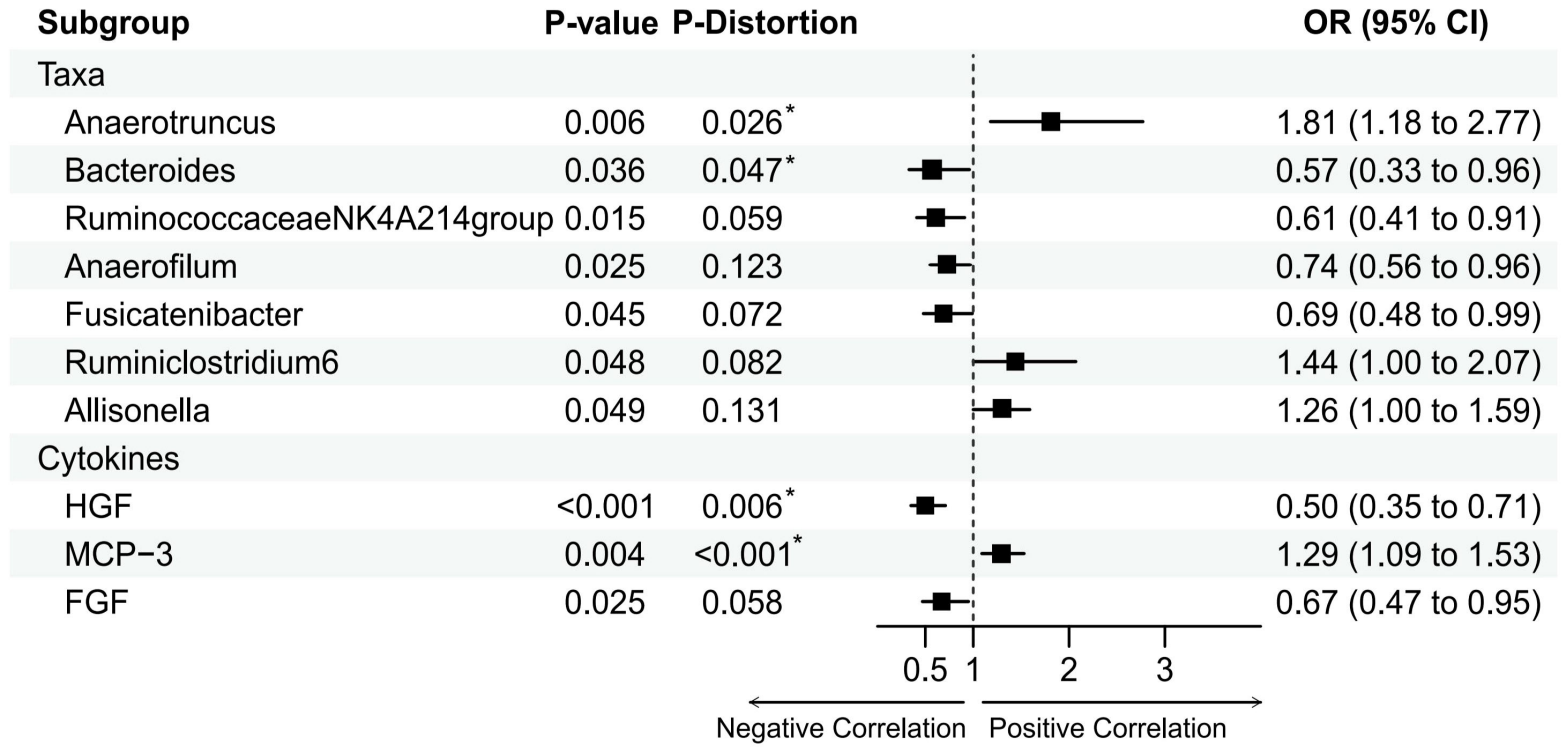
Mendelian randomization analysis of the casual effect of gut microbiota and inflammation cytokines on male infertility. Forest plots showing causal estimates of the association between gut microbiota and male infertility, Odds ratios and 95% confidence intervals were obtained using inverse variance weighted method. P-distortion was obtained using MR-Presso.

Based on the IVW method, we identified seven gut microbiota that may be associated with MI (**Figure 2**). The most significant result was for Anaerotruncus species with an odds ratio (OR) of 1.81 and a 95% confidence interval (CI) of 1.18 - 2.77 (P = 0.0062).

Besides, Anaerofilum(OR = 0.74, 95%CI = 0.56-0.96, P = 0.0249), Ruminiclostridium6(OR = 1.44, 95% CI = 1.00–2.07, P = 0.0476), Allisonella (OR = 1.26, 95% CI = 1.00- 1.59, P = 0.0486), Bacteroides (OR = 0.57, 95% CI = 0.33-0.96, P = 0.0363), Fusicatenibacter(OR = 0.69, 95% CI = 0.48-0.99, P = 0.0449), and RuminococcaceaeNK4A214group (OR = 0.61, 95% CI = 0.41-0.91, P = 0.0153) also had a suggestively causal effect on MI. However, in the MR-Presso distortion test, five of the above seven gut microbiota had P-values that were not significant after being corrected for Allisonella, Ruminiclostridium6, Anaerofilum,Fusicatenibacter, and RuminococcaceaeNK4A214group((see **Supplementary Table 5**). In addition, no inverse causal relationship was found between these seven gut microbiota and MI by MR-Steiger test(see **Supplementary Table 4**).

### 3.2 Casual relationship between inflammation cytokines and male infertility

Based on the IVW method, we identified three inflammatory cytokine that may be associated with MI (**Figure 2**). The most significant result was for Hepatocyte growth factor(HGF) with an OR of 0.50 and a 95% confidence interval (CI) of 0.35-0.71 (P = 0.0001). This result was supported by the MR sensitivity analysis with MR-Presso distortion test (see **Supplementary Table 7**). Besides, Monocyte chemotactic protein 3(MCP-3)(Beta = 0.25, OR = 1.44, 95% CI = 1.00–2.07, P = 0.0039) and Fibroblast growth factor(FGF)(Beta = -0.40, OR = 0.61, 95% CI = 0.41-0.91, P = 0.025) also had a suggestively causal effect on MI. However, the MR-Egger and IVW methods for FGF had effects in opposite directions in the sensitivity analyses, making the causal relationship between FGF and MI unreliable. In addition, no inverse causal relationship was found between these inflammatory cytokine and MI by MR-Steiger test (see **Supplementary Table 6**).

### 3.3 Casual relationship between gut microbiota and inflammatory cytokine

Gut microbiota causally associated with MI and inflammatory cytokines causally associated with MI were analyzed by MR. The IVW method found a positive correlation between the level of Bacteroides and the level of HGF (Beta = 0.32, P= 0.010). This result remained significant after MR-Presso correction (P = 0.027).

### 3.4 Multivariate Mendelian randomization

MVMR results showed that the causal effects of HGF and Bacteroides on MI remained significant when adjusted for HGF or Bacteroides. After adjustment, their causal effects on MI were respectively: HGF(OR = 0.55, 95%CI = 0.31-0.98, P = 0.04),Bacteroides (OR = 0.69, 95%CI= 0.51-0.95, P = 0.02).

### 3.5 The mediation effect of HGF in the causal association between Bacteroides and MI

**Figure 3:**
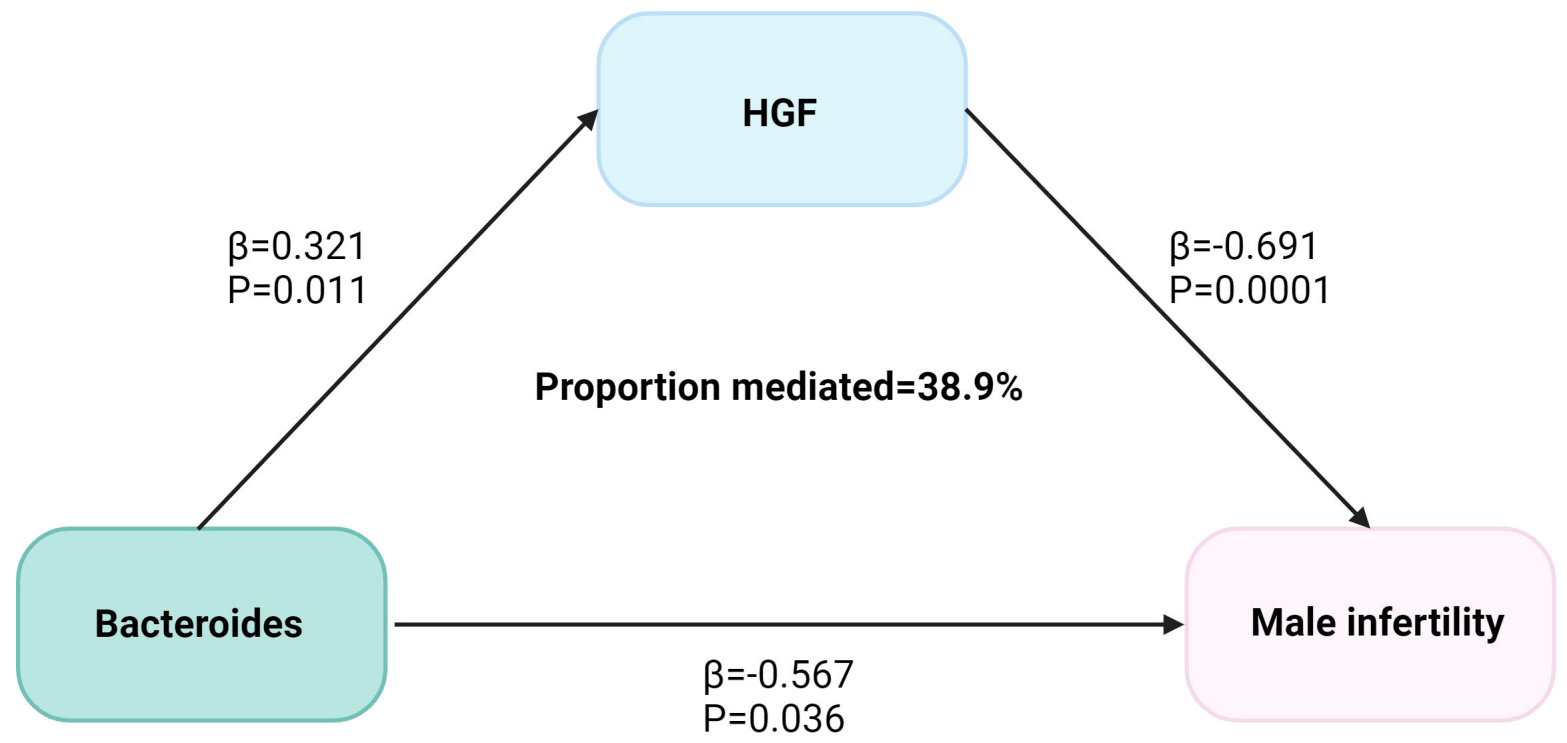
The HGF mediated the causal effect of Bacteroides on male infertility. The β-value was calculated by the inverse variance weighting method, and the mediator ratio was calculated by the formula: β1 × β2/β3. β1 represents the effect value of Bacteroides on HGF, β2 represents the effect value of HGF on male infertility, and β3 represents the effect value of Bacteroides on male infertility.

We analyzed the causal effect of Bacteroides on HGF and depicted the causal link to highlight the mediation role of HGF in the causal relationship between species Bacteroides and MI in **Figure 3**. The Bacteroides causally increased HGF (Beta = 0.321, P = 0.011) and subsequently associated with an increased risk of MI. The indirect effect estimate was Beta = 0.222 (95% CI = 0.043-0.46, P = 0.05) with a mediated proportion of 38.9%.

## 4 Discussion

The present MR analysis study found a causal relationship between two gut microbiota, including Bacteroides, and male infertility. In addition, HGF may have mediated 38.9% of the causal effect of Bacteroides on male infertility. Our MR analysis between gut microbiota, inflammatory cytokines, and male infertility provides evidence for a causal relationship between gut microbiota and male infertility and the role of hepatocyte growth factor as a mediator. In addition, in the MVMR analysis, we found positive results for both Bacteroides and HGF, which may indicate the existence of two scenarios: (1) Exposure factors affect the results not only directly but also indirectly through the mediating factors. In this case, the mediator may be a pathway through which the exposure factor affects the results, but there are other direct pathways through which the exposure factor acts. (2) Exposure and the mediator may each be independently associated with the outcome. This means that, in addition to how the exposure factor affects the outcome through the mediator, the middle factor itself may also affect the outcome through different mechanisms.

16s rRNA gene sequencing is currently the most common method for determining the proportions of various intestinal bacterial groups. Nonetheless, the intestinal microbiota encompassed in the majority of studies is incomplete, and there is poor reproducibility of the inconsistent microbial groups in male infertility patients among different studies. For example, Cao et al. (35) noted a notable decline in Bacteroides in the male infertility cohort, whereas Zhang et al. (36) observed no significant disparity in Bacteroides between the healthy and male infertility cohorts. This discrepancy may be due to different statistical methods or insufficient control of potential confounding factors. Moreover, MR analysis can minimize the result errors caused by the influence of confounding factors.

Several studies have reported associations between the gut microbiota and reproductive capacity in male mammals. The abundance of Anaerotruncus was significantly increased in mice with testicular damage due to diabetes but decreased significantly after treatment, which is consistent with the analyses in this study (37). Alginate oligosaccharides may improve sperm quality by increasing the abundance of anaerobic bacteria and attenuating the abundance of mucus spirochetes in the gut microbiota (38). In addition, an observational study noted a significant decrease in Mycobacterium avium spp. in a male infertility cohort (35). In animal models of metabolic syndrome, the abundance of the Ruminococcaceae_NK4A214_group is positively correlated with bile acid levels (39). When the abundance of the Ruminococcaceae_NK4A214_group decreases, bile acid levels also decline, which impedes the absorption of vitamin A, thereby leading to abnormalities in sperm (39). These findings are consistent with the results obtained in the present study. Research also indicates that the gut microbiome can enhance the blood-testis barrier and maintain normal testosterone levels, thereby preserving male fertility (40).

Previous studies have shown that HGF and its receptor c-MET are closely related to male reproduction. Research results show that HGF is related to testicular development and sex hormone secretion and that HGF can partially affect sperm motility (41). Several studies in mouse animal models have shown that HGF can mediate testicular differentiation and testicular cord formation under ex vivo organ culture conditions (42–44). Another *in vitro* cell experiment showed that HGF can control the mitotic activity of germ cells and significantly increase the proliferation of rat testicular spermatogonia (45). At the same time, HGF has also been proven to significantly reduce germ cell apoptosis and restore spermatogenesis (46). Several studies above have shown that HGF can affect testicular morphology and germ cell production and apoptosis. These factors are closely related to male infertility. However, the above research only stays at the stage of animal models and has certain limitations.

Metagenomic sequencing studies have established associations between the gut microbiome and inflammatory cytokines have been established through metagenomic sequencing studies. Gut microbes and their metabolites alter levels of various inflammatory cytokines (47–49). A study has shown that chronic alcohol consumption disrupts the gut microbial environment, leading to significant decreases in the levels of some of these key gut microbes (e.g., Helicobacter, a genus of Bacteroides), which in turn alters the body’s microenvironment, resulting in altered levels of inflammatory cytokines in the body, and ultimately an increased risk of infertility (50). Despite existing knowledge linking the gut microbiome to inflammatory cytokine levels, the exact mechanisms by which the gut microbiome affects inflammatory cytokine levels, especially HGF, remain unclear. Further exploration of this area has the potential to deepen our understanding of the role of the gut microbiome in the development of MI. Our study shows that the gut microbiota may be a promising strategy for preventing and treating MI. This strategy may modulate the levels of intestinal microbiota and promote microenvironmental balance through probiotic intake, drugs, and bioactive metabolites (51). However, translating existing research into clinically beneficial results still requires further research to understand the mechanism by which intestinal microbiota affects MI and the mediators that play a role.

The strengths of our study are twofold. First, Mendelian randomization analyses can reduce the effects of confounding factors and provide a more robust causality assessment than observational studies. Second, compared with previous Mendelian randomization studies on gut microbiota and male infertility, we enhanced the robustness of our results through the MR-Presso distortion test and sensitivity analyses.

Limitations of our study also need to be acknowledged. First, MR analysis provides a good way to verify the causal relationship between the two. However, MR analysis reflects lifelong genetic exposure, not short-term effects. This may not reflect the benefits of short-term changes in the gut microbiota. However, it can still inform us about the potential direction of effects, which needs to be verified in further studies. In addition, since there is only one GWAS study on male infertility, we could not be validate the MR analysis results using other GWAS data sets. Finally, since the GWAS data used in this study come from European populations, caution should be exercised when generalizing the results of this study to a broader population. Based on the above limitations of this study, future studies could conduct more GWAS on male infertility. Expanding the genetic tools used for Mendelian randomization analyses could improve the robustness of the findings and allow for a more comprehensive exploration of the role of the gut microbiota in male infertility. Further *in vitro* and *in vivo* studies could be conducted to elucidate the pathways by which HGF and other potential mediators affect male reproductive health. Mechanisms of the gut-reproductive system axis can also be further investigated to explore targets for further interventions, thus providing direction for future drug therapy. It is hoped that these limitations can be improved in subsequent GWAS studies.

## 5 Conclusion

This study identified a causal relationship between gut microbes, inflammatory cytokines, and MI. Specifically, Anaerotruncus, Bacteroides, HGF, and MCP-3 had a causal relationship with MI. Furthermore, this study also found that HGF is a mediator between Bacteroides and MI, with a mediation proportion of 38.9%. Our study provides a novel perspective on the mechanisms of the gut-reproductive system axis. Based on the results of this study, future research can further investigate the mechanisms of the gut-reproductive system axis and explore potential intervention targets, thereby offering directions for future pharmacological treatments.

## Data availability statement

The original contributions presented in the study are included in the article/**Supplementary Material**. Further inquiries can be directed to the corresponding author.

## Ethics statement

Ethical approval was not required for the study involving humans in accordance with the local legislation and institutional requirements. Written informed consent to participate in this study was not required from the participants or the participants’ legal guardians/next of kin in accordance with the national legislation and the institutional requirements.

## Author contributions

HZ: Writing – original draft, Visualization, Methodology, Data curation, Conceptualization. NX: Writing – original draft, Methodology. HX: Writing – original draft, Data curation. XX: Writing – review & editing, Conceptualization. YC: Writing – review & editing, Project administration, Methodology. SW: Writing – review & editing, Supervision, Resources, Funding acquisition.
